# Thermal physiological traits in tropical lowland amphibians: Vulnerability to climate warming and cooling

**DOI:** 10.1371/journal.pone.0219759

**Published:** 2019-08-01

**Authors:** Rudolf von May, Alessandro Catenazzi, Roy Santa-Cruz, Andrea S. Gutierrez, Craig Moritz, Daniel L. Rabosky

**Affiliations:** 1 Department of Ecology and Evolutionary Biology, Museum of Zoology, University of Michigan, Ann Arbor, MI, United States of America; 2 Museum of Vertebrate Zoology, University of California, Berkeley, Berkeley, CA, United States of America; 3 Department of Biological Sciences, Florida International University, Miami, FL, United States of America; 4 Área de Herpetología, Museo de Historia Natural de la Universidad Nacional de San Agustín (MUSA), Arequipa, Perú; 5 Facultad de Ciencias Biológicas, Universidad Nacional Agraria La Molina, Lima, Perú; 6 Centre for Biodiversity Analysis and Research School of Biology, The Australian National University, Canberra, Australia; Universitat Trier, GERMANY

## Abstract

Climate change is affecting biodiversity and ecosystem function worldwide, and the lowland tropics are of special concern because organisms living in this region experience temperatures that are close to their upper thermal limits. However, it remains unclear how and whether tropical lowland species will be able to cope with the predicted pace of climate warming. Additionally, there is growing interest in examining how quickly thermal physiological traits have evolved across taxa, and whether thermal physiological traits are evolutionarily conserved or labile. We measured critical thermal maximum (CT_max_) and minimum (CT_min_) in 56 species of lowland Amazonian frogs to determine the extent of phylogenetic conservatism in tolerance to heat and cold, and to predict species’ vulnerability to climate change. The species we studied live in sympatry and represent ~65% of the known alpha diversity at our study site. Given that critical thermal limits may have evolved differently in response to different temperature constraints, we tested whether CT_max_ and CT_min_ exhibit different rates of evolutionary change. Measuring both critical thermal traits allowed us to estimate species’ thermal breadth and infer their potential to respond to abrupt changes in temperature (warming and cooling). Additionally, we assessed the contribution of life history traits and found that both critical thermal traits were correlated with species’ body size and microhabitat use. Specifically, small direct-developing frogs in the Strabomantidae family appear to be at highest risk of thermal stress while tree frogs (Hylidae) and narrow mouthed frogs (Microhylidae) tolerate higher temperatures. While CT_max_ and CT_min_ had considerable variation within and among families, both critical thermal traits exhibited similar rates of evolutionary change. Our results suggest that 4% of lowland rainforest frogs assessed will experience temperatures exceeding their CT_max_, 25% might be moderately affected and 70% are unlikely to experience pronounced heat stress under a hypothetical 3°C temperature increase.

## Introduction

Climate change is affecting biodiversity and ecosystem function worldwide, and the lowland tropics are of special concern because organisms living in this region experience temperatures that are already close to their upper thermal limits [1–4]. However, it remains unclear how and whether tropical lowland taxa will be able to cope with the predicted pace of climate warming. Given that lowland rainforest habitats are expected to become hotter in the coming decades [5–6], it is imperative that we obtain baseline data on critical thermal limits of lowland rainforest species. To this end, measuring physiological thermal limits such as critical thermal maxima (CT_max_) will improve our predictions of species’ vulnerability to climate warming. Furthermore, measuring critical thermal minima (CT_min_) is also important because it will allow us to estimate species’ thermal breadth (= CT_max_−CT_min_) and infer their potential to respond to extreme temperature fluctuations (warming and cooling). Although CT_max_ and CT_min_ measure two very different physiological end points to thermal performance curves (i.e., death occurring at temperatures immediately above CT_max_, but typically not below CT_min_), the ability of organisms to perform basic movements such as the righting reflex is very likely associated with individual fitness.

There is growing interest in examining whether physiological traits are evolutionarily conserved or labile, because knowing how quickly thermal physiological traits evolved can be used to improve predictions on species’ potential to respond to novel climates [7–8]. Growing evidence suggests that CT_max_ is relatively inflexible across elevation (e.g., [8–10], but see [11]), with a narrow upper limit and low plasticity [12–13], and that it is evolutionary stable across a variety of ectotherms [14]. Consequently, one would predict that species living at the same elevation and experiencing the same thermal environment, such as those in lowland tropical rainforest, exhibit narrow upper thermal limits. While lowland taxa may experience environmental temperatures that are closer to their critical thermal limits, not all species in a lowland rainforest community may exhibit similarly narrow upper thermal limits. Likewise, not all species in a lowland rainforest community may be equally vulnerable to increased temperatures (as it would be predicted using bioclimatic variables, e.g., WorldClim [15]). If CT_max_ varies broadly among lowland species, one would infer that only a subset of the species in the community (i.e., those with lower CT_max_ values) is vulnerable to warming.

Another reason to study critical thermal limits pertains to the role of temperature in amphibian immunity and disease dynamics. Previous research has shown that frogs’ immune system might be more effective at deterring pathogen infection (e.g., chytridiomycosis) at warm or more stable temperatures than at cold and variable temperatures [16–18]. In western Amazonia, sudden drops in air temperature associated with incursions of cold air masses coming from southern South America and the Antarctic region are common [19–20]. These cold surges, locally called *friajes* (Peru), *friagen* (Brazil), or *surazos* (Bolivia), are common during the Austral winter and reduce animal activity in lowland Amazonia. Fewer mammals and birds are active during these periods [21–22] and some amphibians behaviorally adjust their vertical distribution along the forest habitat [23]. Additionally, these cold surges may facilitate pathogen infection, such as chytridiomycosis, which is common in some lowland Amazonian habitats [24]. How species respond to lower temperatures during a cold front might depend on their physiological thermal limits. If the temperature drops to 10–12°C, as often does during a cold front [20, 25], many lowland taxa may reach their CT_min_. As a result, these organisms may stop moving and may become vulnerable to predators that tolerate colder temperatures. In turn, species that tolerate cooler temperatures and remain active during a cold front may be able to do so by maintaining a higher metabolic rate, which requires higher use of energy reserves or higher caloric intake [26]. Additionally, given that temperature decreases at a relatively constant rate with increasing elevation (a result of adiabatic cooling), measuring tolerance to cold in lowland species can be useful for inferring their ability to shift to higher elevations.

Here we examined the variation of CT_max_ and CT_min_ in 56 species of lowland Amazonian rainforest frogs to test if tolerance to heat and cold are phylogenetically conserved or labile. We used a phylogenetic framework to account for non-independence of interspecific data resulting from shared ancestry. While we tested if tolerance to heat and cold are phylogenetically conserved or labile, we do not conclude that these traits are exclusively adaptive. Additionally, we assessed whether species are vulnerable to predicted warming trends across the Amazonian lowlands and comment on species’ vulnerability to low temperatures associated with cooling events that are common in the region. We estimated the operative warming tolerance by subtracting the maximum operative temperature from CT_max_, as done in previous studies [11, 27]. Our specific goals were to assess (1) the extent of phylogenetic conservatism in heat and cold tolerance, (2) to determine whether heat-tolerance or cold-tolerance evolves more quickly among species, and (3) to determine whether key life history or morphological attributes predict variation in CT_max_ and CT_min_ across species. We used a phylogenetic comparative framework for all analyses. Using a hypothetical 3°C warming increase (e.g., IPCC warming scenarios RCP6.0 and RCP8.59 [28]), we predict the impacts of future climate warming on this diverse community of rainforest frogs.

## Material and methods

### Study area

We studied amphibian communities at Los Amigos Biological Station (12°34'07" S, 70°05'57" W, 250 m elev.), located in the Madre de Dios region, Peru, between 2012 and 2017. The lowland rainforests of this region contain 85 species of frogs in broad sympatry, and the humid lowlands of western Amazonia include some of Earth’s most species-rich amphibian communities. We previously described the study site, local climate, and amphibian fauna [29–30]. We obtained data on species’ elevational distributions from surveys conducted along the elevational gradient from Los Amigos Biological Station [11, 29–31] to Tres Cruces at 3,800 m [27, 32–34]. Daily temperatures at Los Amigos showed that maximum air temperatures in 2015–2017 were consistently higher than average since recording started in 2000 (e.g., maximum air temperatures of 30°C or higher were very common between November 2015 and April 2016 and 13 days had maximum air temperatures above 38°C [24]).

### Environmental temperatures

We used data loggers to obtain empirical data on microhabitat temperatures from lowland Amazonian habitats. We placed iButton data loggers (Maxim Integrated Products, Sunnyvale, California, USA) in two forest microhabitats, leaf-litter and understory vegetation, used by frogs across forest types. Daily temperatures were recorded in floodplain forest, terra firme forest, bamboo forest, and palm swamp during part of the wet season of 2008 (44 days), part of the wet season of 2016 (25 days), and part of the wet season of 2017 (24 days). Additionally, we placed HOBO data loggers (Onset Computer Corporation, Pocasset, Massachusetts, USA) in the leaf-litter in the floodplain and the terra firme forest to record the temperature from 16 November 2016 to 5 December 2017 (385 days). Daily temperatures measured in these forest types are summarized in [24] and in the Results section (see Temperature data).

### Critical thermal limits

We measured CT_max_ in 384 individuals (56 species) and CT_min_ in 137 individuals (41 of the 56 species). We measured snout-vent length (SVL) to the nearest 0.1 mm with a Vernier caliper, and measured body mass to the nearest 0.1 g using a Pesola scale. Frogs included in our dataset (S1 Appendix) had an SVL range of 10.1–51.0 mm; we excluded large-bodied species (i.e., species exhibiting maximum outlying values of SVL and body mass). We captured animals in the field and transported them to a field laboratory, where we kept them in individual containers with a thin layer of water embedded in a paper towel for 2–3 days prior to measurements. Plasticity and adaptation likely influence the traits we measured, because we performed our experiments in the field where fully controlled conditions are difficult to achieve. Nevertheless, we strived to maintain similar ambient temperature and acclimation conditions for all taxa (within 24–26°C range). We used non-lethal experiments to evaluate critical thermal maxima (CT_max_) and minima (CT_min_). We measured CT_max_ and CT_min_ as the point when frogs lost their righting response, defined as the moment when a frog cannot right itself from being placed venter-up for a period longer than 5 sec [27, 35]. The righting response is relevant for considering selection on thermal physiology, because a frog that is unable to display their automatic righting reflex will likely be unable to escape predators. We placed each individual in a plastic cup with a thin layer of water (3–5 mm) and immersed the cups in a water bath. For CT_max_, we increased the bath temperature from ~24°C to up to ~40°C at a rate of ~1°C/minute by adding warm water. For CT_min_, we decreased the temperature from ~24°C to ~0°C by adding ice to the water bath [36]. We forced animals to a venter-up position, and we used a quick-reading thermometer to measure temperature against the body of the frog immersed in the thin layer of water. In this procedure, the tip of the thermometer should be placed between two skin surfaces (e.g., groin region) so that most of it is in contact with the animal. We ended the experiments when the animals were unable to right themselves for >5 sec (i.e., when they lost their righting response). We included as controls individuals of representative species (N = 29 individuals, 14 species) that were similarly probed for their righting reflex over ~20 minutes, but without experiencing temperature change, to take into account any potential behavioral or fatigue effect [37]. No individuals lost their righting reflex during these control trials.

After experimentation, animals were released at the point of capture and only a few individuals were euthanized by immersion in benzocaine hydrochloride solution (250 mg/L), where animals were kept for 10–20 minutes until movement ceased. After euthanasia, tissue samples (e.g., liver, muscle) were taken from the animals and preserved in 2 mL cryogenic tubes filled with RNAlater or 95% ethanol. Following tissue collection, specimens were fixed in 10% formalin, and permanently stored in 70% ethanol. Voucher specimens were deposited in the Herpetological Collections of the University of Michigan Museum of Zoology (UMMZ) and the Museo de Historia Natural of the Universidad Nacional Mayor de San Marcos (MUSM) in Peru.

Given the small size of the frogs included in this study (range 10.1–51.0 mm in SVL), we assumed that this temperature is equivalent to the core temperature of frogs [35]. While we did not measure individuals’ body temperatures with thermocouple probes inserted into the frogs’ cloaca, it is reasonable to assume that our measurements (taken with a quick-reading thermometer placed against the body of the frog) reflect body temperatures. We justify this assumption on (*i*) empirical evidence shows that cloacal temperature and skin temperature are strongly correlated [38] and represent the core temperature; (*ii*) paired tests using live frogs reaching a maximum SVL of 21 mm and similarly-sized plaster models suggest that individual frogs achieve equilibrium body temperature within 1 min [39]; and, (*iii*) differences in heating rates (e.g., 1°C/1 min vs. 1°C/5 min) in individuals larger than 10 mm do not result in different heat tolerances [40]. Our empirical data also supported the assumption that the body size of experimental individuals did not bias our measurements of critical thermal limits (see S1 File and S1 Fig, S2 Fig, S3 Fig and S4 Fig). Thus, differences in critical thermal traits among species, such as those reported here, likely reflect biological differences.

### Phylogenetic data

Our analysis included DNA sequences from three mitochondrial genes (12S, 16S, COI) and two nuclear genes (RAG-1, Tyr). Extraction, amplification, and sequencing of DNA followed protocols described previously [11] (see also S2 File). We used a multispecies coalescent approach implemented in *BEAST 2 [41] to infer a Bayesian multilocus timetree of the focal taxa. The primary goal of the analysis was to obtain an ultrametric tree used in phylogenetic comparative analyses (see below). Our analyses depended on the relative branch lengths of the tree, but we preferred to illustrate our tree in rough units of time. Thus, we used an uncorrelated relaxed molecular clock with the rate of nucleotide substitution for 16S set at 1% per million years as done in recent studies [11, 42]. However, we note that the dates associated with the tree should only be viewed as approximate and that there are multiple sources of error when calibrating phylogenies [43]. The tree included sequence data used in previous analyses [44] and sequence data obtained from specimens collected in the study region (S2 Appendix). The analysis in *BEAST included two independent runs, each with 1 billion generations and sampled every 100000 generations. Following the completion of the analysis, we used Tracer v1.5 [45] to examine effective sample sizes, verify convergence of the runs, and to ensure the runs had reached stationarity. Observed effective sample sizes were sufficient for most parameters (ESS >200) except for substitution rates for a few partitions. We discarded the first 10% of samples from each run as burn-in. Subsequently, we used LogCombiner to merge all remaining trees from both runs and used TreeAnnotator [45] to summarize trees and obtain a Maximum Clade Credibility tree.

### Phylogenetic signal

For a given quantitative trait, phylogenetic signal is present when related species tend to resemble one another [46–47]. We tested for phylogenetic signal by calculating the K statistic [47] and by estimating the λ parameter [48]; we used the R package ‘phytools’ [49] to estimate K and λ. These methods account for non-independence of interspecific data resulting from shared ancestry [50–52]. For K, values smaller than 1 indicate that related species are less similar than expected under a Brownian motion model of trait evolution whilst values greater than 1 indicate that related species resemble each other more than expected under a Brownian motion model of trait evolution [47]. The value of λ typically ranges from 0, indicating no phylogenetic signal, to 1, indicating strong phylogenetic signal (i.e., when patterns of covariance among species are exactly as predicted under a Brownian motion model of evolution [48]).

### Rates of evolutionary change in critical thermal traits

Prior to comparing the rates of evolutionary change for CT_max_ and CT_min_, we searched for a model of evolution that best explains the variation in the observed data. We used the fitContinuous function in GEIGER [53] to fit three models of evolution: Brownian Motion (BM), single-optimum Ornstein-Uhlenbeck (OU), and Early Burst (EB). The Brownian motion model assumes a zero net change, but the underlying evolutionary process has a constant variance per unit time, and the differences between species will be proportional to the time since their divergence. The Ornstein-Uhlenbeck model describes a stochastic process involving an overall global optimum, but with a restraining parameter that determines the intensity of attraction between a particular trait value and the optimum. With OU, differences between species will not necessarily relate to their time since divergence. Finally, the Early Burst model assumes an exponential decline in rates through time. This means that species with recent divergence times will be very similar, while species with deeper divergences will be proportionately more dissimilar than closely related lineages. After determining the best fitting model of evolution for each trait, we used the R package ‘APE’ [54] and code developed by Adams [55] to estimate the rates of change.

### Correlates of CT_max_ and CT_min_

We explored the relationship between critical thermal traits and other life history characteristics including body size (SVL), body mass, and body mass index (BMI). We calculated BMI as the ratio of weight to size by dividing the mass (in g) by the square of SVL (in mm). We used analysis of covariance (ANCOVA) to test the effect of family membership on both CT_max_ and CT_min_ while controlling for the effect of SVL (co-variable). We also considered maximum air temperatures (T_a_) obtained from a local weather station, and maximum operative temperatures (T_e_) estimated from field measurements taken with data loggers placed in the forest floor and understory vegetation in mature floodplain forest and terra firme forest. We calculated operative warming tolerance (OWT) by subtracting the average maximum T_e_ from CT_max_ as in previous studies [11, 27]. In addition to OWT, we used bioclimatic data from WorldClim [15] to calculate warming tolerance (WT_w_) as in [56]; we calculated WT_w_ by subtracting the maximum temperature of the warmest month (bioclimatic variable BIO6 in WorldClim [15]) from CT_max_. Lastly, we calculated the thermal breadth, defined as the difference between CT_max_ and CT_min_. We examined a pairwise scatterplot matrix to visualize the cross-correlations among variables (S5 Fig) and discarded predictor variables that were highly correlated with each other (r > 0.70). We used the R package ‘phylolm’ [57–58] to fit phylogenetic generalized linear regression models (PGLMs). This package implements a phylogenetic regression under various models for the residual error, including Brownian Motion (BM) and Ornstein-Uhlenbeck (OU). We used the AIC value to identify the model that best explains the variation of observed data [58].

We estimated the relative importance of each variable in explaining the observed variation of CT_max_ and CT_min_ with multiple regression [59]. First, we used the pairwise scatterplot matrix (S5 Fig) and discarded variables that were highly correlated (r > 0.70). Then, we ran PGLMs for all possible additive models including the predictor variables. We calculated the Akaike weight for each model, and the relative importance of each factor was inferred by examining the extent of its contribution to highly scoring models. We used the sum of the relative Akaike weights for the models containing that factor to determine its relative importance.

## Results

### Temperature data

Data recorded over a 1-year period (16 Nov. 2016–5 Dec. 2017) indicate that daily minimum, mean, and maximum temperatures measured in the leaf litter of the floodplain and terra firme forests were similar (S6 Fig). Daily maximum temperatures were similar most of the year, with the exception of some periods of higher maximum temperature in the floodplain than in the terra firme forest. During this year, there were two pulses of high temperature reaching 34.8 and 35.1°C, respectively, in the floodplain forest. These two pulses were four days apart and the intermediate days had maximum temperatures ranging from 29.5 to 31.8°C. However, the pulse with the highest temperature (35.1°C) was followed by four days with maximum temperatures above 33.3°C; this warming event took place between 29 August and 2 September 2017. During this period, minimum temperatures in the floodplain ranged between 23.3 and 24.0°C and minimum temperatures in the terra firme ranged between 23.5 and 24.2°C. Furthermore, there were two pulses of high temperature reaching 28.4 and 29.2°C, respectively, in the terra firme forest. However, these pulses occurred in different dates than those observed in the floodplain forest. Despite these differences in observed temperature in the two forest types, maximum daily temperatures in the leaf litter remained <28.0°C throughout most of the year, with average maximum temperature of 26.8°C. During this year, the lowest temperatures were 13.4°C in the floodplain and 13.9°C in terra firme. However, minimum temperatures in previous years were lower and similar to those recorded throughout the western Amazon (10–12°C [19, 25]).

### Phylogenetic relatedness and critical thermal traits

**Fig 1:**
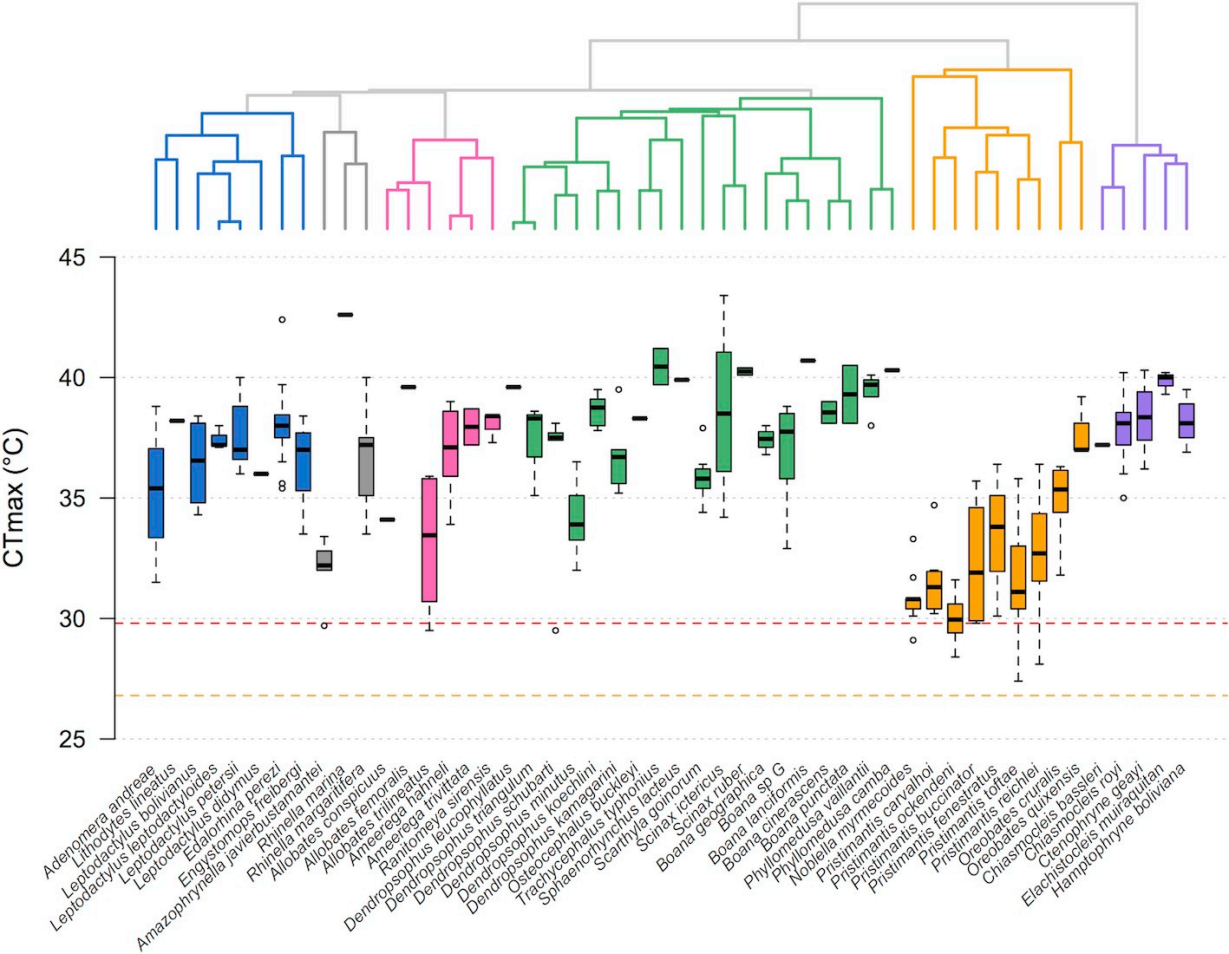
Divergence in CT_max_ in lowland Amazonian frogs. Multi-locus tree depicting the relationship among 50 species (top) and box plots depicting their CT_max_ values (bottom). The box plots show the median (black bar), interquartile range (box), and 1.5 times the inter-quartile range (bars); circles represent outliers. Species are color-coded according to family membership (blue = Leptodactylidae, gray = Bufonidae, pink = Dendrobatidae, green = Hylidae, orange = Strabomantidae, violet = Microhylidae). The orange line (dashes) represents the estimated maximum temperature near the forest floor, measured with iButton data loggers; the red line (dashes) represents a hypothetical 3°C increase in temperature.

**Fig 2:**
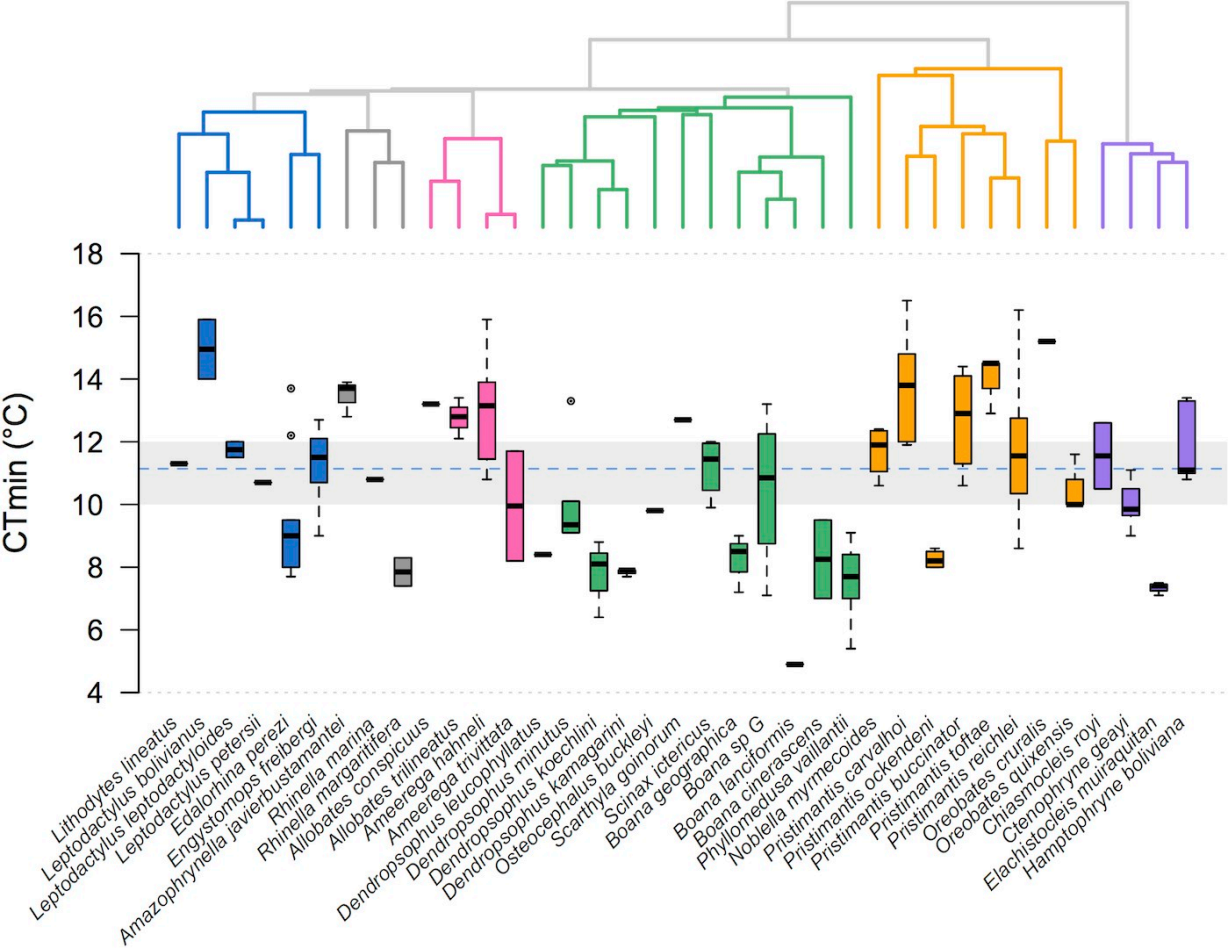
Divergence in CT_min_ in lowland Amazonian frogs. Species tree depicting the relationship among 37 species (top) and box plots depicting their CT_min_ values (bottom). The box plots show the median (black bar), interquartile range (box), and 1.5 times the inter-quartile range (bars); circles represent outliers. Species are color-coded according to family membership (blue = Leptodactylidae, gray = Bufonidae, pink = Dendrobatidae, green = Hylidae, orange = Strabomantidae, violet = Microhylidae). The gray area represents the range of typical cold front minimum temperatures (10–12°C [20, 25]) and the blue line represents the average temperature recorded during cold fronts between 2001 and 2017.

We observed considerable differences in CT_max_ values (27.4–43.2°C; Fig 1). Eight pairs of close relatives had non-overlapping CT_max_ values (*Adenomera andreae*–*Lithodytes lineatus*, *Rhinella marina*–*R*. *margaritifera*, *Allobates conspicuus*–*A*. *femoralis*, *Dendropsophus leucophyllatus*–*D*. *triangulum*, *Dendropsophus schubarti*–*D*. *minutus*, *Dendropsophus koechlini*–*D*. *kamagarini*, *Phyllomedusa vaillantii*–*P*. *camba*, *Elachistocleis muiraquitan*–*Hamptophryne boliviana*). *Rhinella marina* (the cane toad, distributed in lowlands and lower montane forests < 1200 m) had the highest CT_max_, whereas several members of the family Strabomantidae (*Noblella myrmecoides*, *Pristimantis buccinator*, *P*. *toftae*, *P*. *ockendeni*) had the lowest CT_max_. CT_min_ varied considerably among species (4.9–16.2°C; Fig 2), and nine pairs of close relatives exhibited non-overlapping CT_min_ values (*Leptodactylus leptodactyloides*–*L*. *petersii*, *Edalorhina perezi*–*Engystomops freibergi*, *Rhinella marina*–*R*. *margaritifera*, *Ameerega hahneli*–*A*. *trivittata*, *Boana* sp. G–*B*. *lanciformis*, *Pristimantis carvalhoi*–*P*. *ockendeni*, *Pristimantis toftae*–*P*. *reichlei*, *Oreobates cruralis*–*O*. *quixensis*, *Elachistocleis muiraquitan*–*Hamptophryne boliviana*).

### Phylogenetic signal

We found moderate phylogenetic signal for CT_max_ (Table 1), as suggested by an overall trend in which closely related species (especially those in families Leptodactylidae, Strabomantidae, and Microhylidae) exhibited more similar CT_max_ than distantly related species (Fig 1). In contrast, no phylogenetic signal was detected for CT_min_ (i.e., close relatives were less similar than expected from Brownian motion along the tree). Additionally, there was no phylogenetic signal for body size (SVL) and body mass index (Table 1).

**Table 1:** Results from the tests for phylogenetic signal based on two statistics, K and λ. Log likelihood values included correspond to the λ estimates. Phylogenetic signal tests were done with the full dataset (50 species) for all traits except for CT_min_. Phylogenetic signal tests were conducted for CT_min_ and repeated for CT_max_ with the reduced dataset (37 species). P-values are relative to the null hypothesis of phylogenetically-unstructured data (see text for details).

Trait	K	P-value	λ	P-value	lnL
*Analyses with full dataset (50 species)*					
CT_max_	0.7480	**0.0010**	0.8212	**<0.0001**	–113.35
SVL	0.4626	**0.0430**	0.5337	0.3602	–184.23
BMI	0.5354	0.4975	0.7134	0.0712	–64.76
*Analyses with reduced dataset (37 species)*					
CT_min_	0.4890	0.1542	0.3954	0.1926	–83.10
CT_max_	0.9116	**0.0010**	1.0296	0.0001	–85.02

### Rates of thermal physiological change

Comparisons across three models of trait evolution indicate that BM was the best model for CT_max_, whereas OU was the best model for CT_min_ (Table 2). Given that the method used for estimating the rates of evolution [55] assumes a zero net change (BM), we performed this test assuming BM for both traits and using the reduced dataset (37 species). We found that CT_max_ and CT_min_ exhibit similar rates of thermal physiological change (likelihood ratio test, LRT = 0.362, AICc = 344.790, P = 0.547).

**Table 2:** Summary table comparing the fit of three models of evolution tested for CT_max_ and CT_min_ data. Likelihood estimates (lnL) and corrected Akaike Information Criterion (AIC_C_) values for tests considering the reduced dataset (i.e., 37 species with both CT_max_ and CT_min_ data) are provided; AIC_C_ values in bold indicate the best-supported model.

	CT_max_ (reduced)		CT_min_ (reduced)	
Model	lnL	AICc	lnL	AICc
Brownian Motion	–85.40	**175.15**	–88.62	181.59
Ornstein-Uhlenbeck	–85.24	177.20	–83.19	**173.11**
Early Burst	–85.40	177.52	–88.62	183.96

### Correlates of CT_max_ and CT_min_

**Fig 3:**
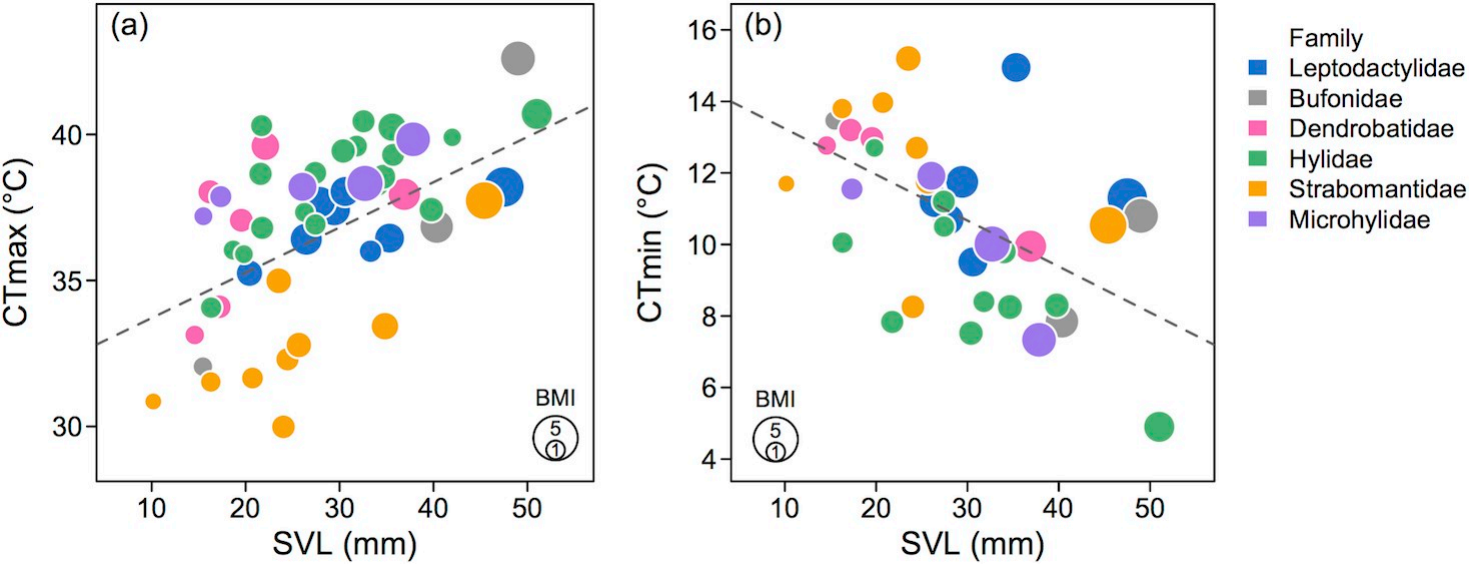
Critical thermal traits are correlated with body size. CT_max_ tends to increase with increasing body size (a) and CT_min_ tends to decrease with increasing body size (b). Species are color-coded according to family membership (see also Fig 1 and Fig 2), and the size of points is proportional to body mass index (BMI). The slope of the regression line reflects the phylogenetic correction in each model, considering the full dataset for CT_max_ (50 species) and the reduced dataset for CT_min_ (37 species).

Interspecific variation in critical thermal limits was correlated with body size. Our PGLS analyses showed that CT_max_ tends to increase with increasing body size while CT_min_ tends to decrease with increasing body size (Table 3; Fig 3). Additionally, CT_max_ negatively correlated with CT_min_ (Table 3), whereas thermal breadth correlated with body size (S5 Fig). All tested relationships were significant under pure OLS regression, the Brownian motion (BM) model, and under the Lambda transform model. Nevertheless, these relationships were best supported under the Lambda model, and, in the case of CT_max_ vs. CT_min_, both the Lambda and BM model provided equal support (i.e., had similar IAC values; Table 3). Most other models ran with two or more variables did not provide a better fit predicting CT_max_ compared to univariate models (i.e., AIC values of models with two or more variables were greater than AIC values of univariate models; S1 Table); the exception to this was a model ran with body size and height above the ground. In contrast, several models ran with two or more variables provided a better fit predicting CT_min_ compared to univariate models (S1 Table). The two best models predicting CT_min_ included (i) a model ran with body size and height above the ground and (ii) a model ran with BMI and height above the ground (S2 Table).

**Table 3:** Results from phylogenetic generalized linear regression models for CT_max_ and CT_min_ and body size (SVL = snout-vent length). Model fitting for CT_max_ included the full dataset (50 species); model fitting for CT_min_ included the reduced dataset (37 species). Bold font indicates significant values.

Model	Evol. model	λ	Coefficient	P-value	AIC	logLik
CT_max_ ~ SVL	OLS		0.1697	**<0.001**	233.70	–112.90
CT_max_ ~ SVL	BM		0.1500	**<0.001**	212.50	–102.20
CT_max_ ~ SVL	Lambda	0.82	0.1550	**<0.001**	207.61	–99.81
CT_min_ ~ SVL	OLS		–0.1132	**0.002**	165.75	–78.87
CT_min_ ~ SVL	BM		–0.1497	**<0.001**	163.45	–77.73
CT_min_ ~ SVL	Lambda	0.77	–0.1286	**<0.001**	161.69	–76.84
CT_max_ ~ CT_min_	OLS		–0.6438	**0.001**	181.75	–86.87
CT_max_ ~ CT_min_	BM		–0.4382	**0.003**	169.20	–80.60
CT_max_ ~ CT_min_	Lambda	1.00	–0.4382	**0.003**	169.20	–80.60

The ANCOVA indicated that both body size and family membership, but not their interaction, affected CT_max_ (Table 4). These results suggest that the slopes of the regression lines between CT_max_ and SVL were similar for all groups (families) considered in this test. Additionally, removing the interaction (between SVL and family) did not affect the fit of the model (F = 1.424, P = 0.2378). Thus, body size had a positive and significant effect on CT_max_, and the effect was similar for all families included in the analysis. Nonetheless, terrestrial breeding frogs (Strabomantidae) had lower CT_max_ than that of other families (Fig 3). The ANCOVA ran on the reduced dataset (37 species) indicated that both body size and family membership, but not their interaction, affected CT_min_ (Table 4). These results suggest that the slopes of the regression lines between CT_min_ and SVL were similar for all groups. Moreover, removing the interaction (between SVL and family) did not affect the fit of the model (F = 0.687, P = 0.638). Thus, body size had a negative and significant effect on CT_min_, and the effect was similar for all families included in the analysis. Hylid frogs (Hylidae) had lower CT_min_ than that of other families (Fig 3).

**Table 4:** Results from analysis of covariance (ANCOVA) used to test the effect of family membership on both CT_max_ and CT_min_ while controlling for the effect of SVL (co-variable). Model fitting for CT_max_ was done with the full dataset (50 species); model fitting for CT_min_ was done with the reduced dataset (37 species). Bold font indicates significant values.

Model	Source	df	Sum of squares	F ratio	P-value
CT_max_ ~ SVL × Family					
	SVL	1	135.92	59.84	**<0.001**
	Family	5	164.96	14.52	**<0.001**
	SVL × Family	5	16.17	1.42	0.238
CT_max_ ~ SVL + Family					
	SVL	1	135.92	57.03	**<0.001**
	Family	5	165.00	13.84	**<0.001**
CT_min_ ~ SVL × Family					
	SVL	1	48.58	14.16	**<0.001**
	Family	5	56.40	3.29	**0.020**
	SVL × Family	5	11.78	0.69	0.638
CT_min_ ~ SVL + Family					
	SVL	1	48.58	14.94	**<0.001**
	Family	5	56.40	3.47	**0.014**

**Fig 4:**
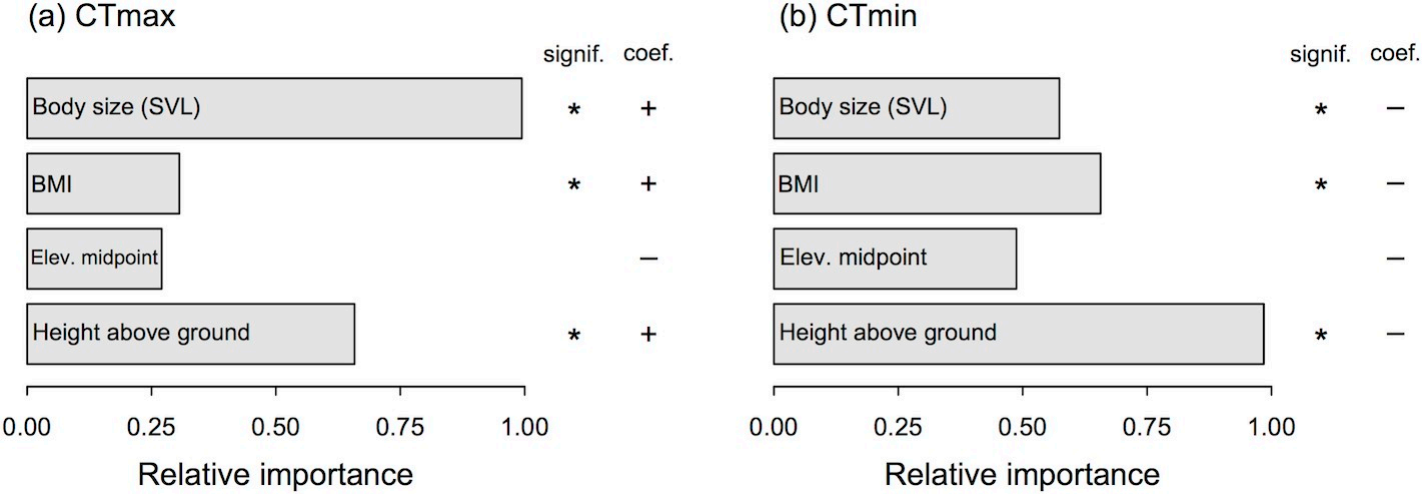
Relative importance of morphological and ecological factors in explaining variation in CT_max_ and CT_min_, based on PGLMs. (a) Model fitting for CT_max_ was done with the full dataset (50 species); (b) model fitting for CT_min_ was done with the reduced dataset (37 species). The bars depict the relative importance of each variable, estimated across additive models and weighted by relative AIC weights, followed by the significance level (asterisk denotes a significant correlation, P < 0.05) and the directionality of the coefficient for each variable.

Our tests using PGLMs and considering several predictor variables suggested that body size was the most important variable for CT_max_, whereas height above the ground was the most important variable for CT_min_ (Fig 4). Additionally, height above the ground was the second most important variable for CT_max_, whereas body mass index was the second most important variable for CT_min_ (Fig 4). Critical thermal traits did not correlate with the elevational midpoint of species. Additional tests also showed that critical thermal traits did not correlate with species’ maximum elevation and elevational range (both of which were correlated with elevational midpoint).

## Discussion

We provide critical thermal trait data for 56 species of lowland Amazonian frog species living in broad sympatry, increasing our knowledge of the thermal physiology of diverse tropical amphibian communities. Previous studies of critical thermal traits in tropical frog assemblages (e.g., [60–61]) included a relatively small number of species present in those assemblages (10–19 species), whereas our study included a larger portion (~65%) of the species present in a diverse Neotropical frog community. One of our key findings is the high variability in both CT_max_ (27.4–43.2°C) and CT_min_ (4.9–16.2°C) in species living in largely undisturbed habitats. The range of CT_max_ values is comparable to that observed in frogs found across a habitat gradient spanning forest to converted habitats such as palm, banana, and pineapple plantations (e.g., [61–62]). In some of these studies, frog species restricted to continuous forest exhibited CT_max_ values (~28°C) that were similar the lowest CT_max_ values reported in our study. However, our study also recovered high CT_max_, previously associated with communities inhabiting disturbed habitats, in frogs inhabiting a largely pristine lowland rainforest.

About one third of the species included in our study exhibited intraspecific variation in CT_max_ and CT_min_ that is worth noting (in both cases, the range was 4–5°C), given that within-population variability is an important (yet underappreciated) attribute of species’ thermal physiology [63–64]. Alternatively, given so much variation, traits may be very plastic and could change quickly even over an individual’s lifetime. Our findings, along with those from a recent study focusing on frogs distributed along a tropical elevational gradient [11], suggest that niche divergence in tolerance to heat and cold is common in tropical ectothermic vertebrates. Additionally, both CT_max_ and CT_min_ exhibited similar rates of evolutionary change despite differing amounts of phylogenetic signal (CT_max_ exhibited a moderate phylogenetic signal and CT_min_ did not exhibit significant phylogenetic signal). Nonetheless, the potential for evolutionary response in CT_max_ might not be sufficient to absorb the rapid warming that is predicted to impact the humid lowland tropics [13]. While all species evaluated here experienced the same (or very similar) thermal regime prior to measurements of CT_max_ and CT_min_, our measures relate to thermal limits under field conditions and are likely influenced by both plasticity and adaptation.

We observed notable interspecific variation in both CT_max_ and CT_min_ for all families, and this trait variation was associated with body size and phylogenetic relatedness among taxa. Body size had a positive and significant effect on CT_max_. Conversely, body size had a negative and significant effect on CT_min_. When considering body size as a covariate, tree frogs (Hylidae) and microhylid frogs (Microhylidae) tolerated warmer temperatures than other taxa. In contrast, direct-developing frogs in the Strabomantidae family appear to be at highest risk of thermal stress. Likewise, in Central America, terrestrial breeding frogs in the Craugastoridae family (which according to some authors includes all species in the Strabomantidae clade) exhibit relatively low CT_max_ and are at risk of thermal stress [62]. Thus, given that converted habitats experience warmer temperatures and broader temperature fluctuations than continuous forest habitat, both groups of terrestrial breeding frogs are susceptible to habitat conversion. This is particularly concerning because both frog families, along with three other families belong to a diverse amphibian clade (Terraranae) containing over 1,065 named species [65].

One important consideration is that CT_max_ and CT_min_ are physiological variables with different ecological implications and, as such, temperatures approaching CT_min_ or CT_max_ have different effects on the activity and survival of organisms. In most ectothermic vertebrates, the lower thermal limit is closely linked to behavior, and individuals experiencing temperatures below CT_min_ may experience inactivity but not necessarily death [63]. In contrast, the upper thermal limit is closely linked to survival and temperatures exceeding CT_max_ may result in death [63]. As a result, the interpretation of thermal breadth should be viewed in light of the potential effect of CT_max_, which anticipates physiological collapse (i.e., death) at one extreme, and the potential effect of CT_min_, which encompasses a broader range of physiological mechanisms (e.g., metabolic downregulation, behavioral impairment, physiological collapse) at the other extreme. In this context, it is not necessarily surprising that CT_min_ is generally more labile than CT_max_ in some clades (e.g., [8, 10]).

Our data suggest that most lowland frog species are not likely to experience critical temperatures as a consequence of modest increases in temperature. Specifically, our comparisons of CT_max_ vs. a hypothetical 3°C temperature increase (Fig 1) suggested that 4% of lowland rainforest frogs assessed will experience temperatures exceeding their CT_max_, whereas 25% might be moderately affected and 70% are unlikely to experience pronounced heat stress. Thus, we predict that most frog species living in continuous, lowland Amazonian forests should be able to tolerate predicted temperature increases. Nonetheless, measuring critical thermal limits at a set temperature does not allow estimation of reaction norms and plasticity of thermal traits within and across species, which are likely to play an important role in species’ response to climatic conditions.

We used CT_max_ and CT_min_ to estimate thermal breadth and infer species’ potential to respond to abrupt changes in temperature, including cooling events such as the cold surges that take place in western Amazonia during the Austral winter. To our knowledge, the lowest ever recorded minimum temperature in Madre de Dios region is 4.5°C, measured in Puerto Maldonado during a cold front in July 1975 [19]. During such extreme cold front, air temperatures remained below 8°C for five days [19]. Only 10 out of 37 frog species we studied (27%) had CT_min_ values around or below 8°C, suggesting that most species (75%) might need to adjust their behavior (e.g., move to lower forest strata, seek thermal refugia in the leaf litter or underground retreat sites) to cope with lower temperatures.

Thermal ecology data from tropical lowland amphibians and other tropical ectotherms continue to be extremely limited [63]. To date, most macroecological models using thermal physiology [4, 12–14] have used data that were primarily collected between the 1960’s and the 1980’s, and were heavily biased towards temperate taxa. Additionally, numerous studies that use macroclimatic data (e.g., WorldClim bioclimatic variables [15]) and species distribution modeling to infer species’ responses to climate change (e.g., [66]) have assumed that species within a given elevation share similar realized niches and might also share similar critical thermal limits. As a consequence, it is assumed that species need to track changes in their environment in order to survive. Many studies using these approaches do not take into account empirical data on species’ critical thermal traits. Given that tropical rainforests contain the most diverse organismal communities on Earth, we need more primary data on species thermal physiology to improve our assessment of species’ vulnerability to climate change. Additionally, further studies should also consider the effect of reduced moisture and water availability, which are major determinants of frog activity and fitness [63].

## Conclusions

A widely held assumption is that organisms living in the same area share similar climatic niches. However, using a phylogenetic framework, our study documents high variability in tolerance to both heat and cold among closely related species living in sympatry. We examined the variation of tolerance to heat and cold in lowland Amazonian rainforest frogs to test if these thermal physiological traits are phylogenetically conserved or labile. Knowing how quickly thermal physiological traits evolved can be used to improve predictions on species’ potential to respond to novel climates. We observed notable interspecific variation in both tolerance to heat and cold, and this trait variation was associated with body size and phylogenetic relatedness among taxa. Our data suggested that thermal physiological traits in lowland frogs are evolutionarily labile and exhibit similar rates of thermal physiological change. Yet, CT_max_ and CT_min_ are physiological variables with different evolutionary implications in relation to climate, and it remains unclear how much variation in CT_max_ is adaptive. We also found that key life history traits have potentially different effect on CT_max_ and CT_min_. Lastly, our data suggest that most lowland frog species are not likely to experience body temperatures exceeding their critical temperature maxima under a hypothetical warming scenario of 3°C increase in air temperature.

## Supporting information

S1 File Relationship between body size and critical thermal limits. (DOCX)

S2 File Further methodological details on molecular phylogenetic analysis. (DOCX)

S1 Table Results from phylogenetic generalized linear regression models to determine which factors best predict variation in CT_max_. Model fitting was done with the full dataset (50 species). Bold font indicates significant values. SVL = snout-vent length, BMI = body mass index, midpoint = elevational midpoint, Height = median height above the ground.
(DOCX)

S2 Table Results from phylogenetic generalized linear regression models to determine which factors best predict variation in CT_min_. Model fitting was done with the reduced dataset (37 species). Bold font indicates significant values. SVL = snout-vent length, BMI = body mass index, midpoint = elevational midpoint, Height = median height above the ground.
(DOCX)

S1 Fig Scatterplots showing the relationship between CT_max_ and SVL. At the intraspecific level, CT_max_ was not correlated with body size in all species tested except *Noblella myrmecoides*.
(TIFF)

S2 Fig Scatterplots showing the relationship between CT_min_ and SVL. At the intraspecific level, CT_min_ was not correlated with body size.
(TIFF)

S3 Fig Scatterplots showing the relationship between CT_max_ and mass. At the intraspecific level, CT_max_ was not correlated with body mass in most species tested.
(TIFF)

S4 Fig Scatterplots showing the relationship between CT_min_ and mass. At the intraspecific level, CT_min_ was not correlated with body mass in most species tested.
(TIFF)

S5 Fig Pairwise scatterplot matrix displaying the relationship between relevant pairs of variables measured in this study. (TIFF)

S6 Fig Daily temperature data. Daily temperatures recorded from 16 November 2016 to 5 December 2017 (385 days) in two forest types at Los Amigos Biological Station, Peru. Weather station data are shown in gray; temperature data collected in the leaf litter are shown in green (floodplain forest) and blue (terra firme forest). In each case, the maximum and minimum temperatures delimited the polygons and the line in the middle represents the mean temperature.
(TIFF)

S1 Appendix Data set analyzed in this study. (XLSX)

S2 Appendix Voucher numbers and GenBank accession numbers for the taxa and genes sampled in this study. (DOCX)
