# Supplementary material for: Thermal physiological traits in tropical lowland amphibians: Vulnerability to climate warming and cooling
Source: PLoS One. 2019 Aug 1;14(8):e0219759. doi: 10.1371/journal.pone.0219759 (PMC6675106; doi:10.1371/journal.pone.0219759)
Supplement: S1 File — (DOCX) [file pone.0219759.s001.docx]

**S1 File. Relationship between body size and critical thermal limits**. We examined the relationship between body size and critical thermal limits to test the assumption that the body size (or body mass) of experimental individuals did not bias our measurements of CT_max_ and CT_min_. We tested this relationship using data from species represented by ≥ 8 measurements of either CT_max_ or CT_min_ in our dataset; we tested the correlations between CT_max_ and SVL (15 species) and between CT_min_ and SVL (4 species). We also used body mass as explanatory variable, and we included data from species represented by ≥ 8 measurements of body mass; we tested the correlations between CT_max_ and mass (15 species) and between CT_min_ and body mass (4 species).

Our tests using body size as explanatory variable showed that, in all but one species, critical thermal limits were not correlated with body size (S1 Fig and S2 Fig). At the intraspecific level, CT_max_ was not correlated with body size in 14 species tested (Table A), whereas CT_max_ was positively correlated with body size in only in one species, *Noblella myrmecoides* (P = 0.044 and Adjusted R^2^ = 0.386). Likewise, at the intraspecific level, CT_min_ was not correlated with body size in all species tested (Table A).

Our tests using body mass as explanatory variable showed that, in most species, critical thermal limits were not correlated with body mass (S3 Fig and S4 Fig). At the intraspecific level, CT_max_ was not correlated with body mass in 12 species out of 15 species tested; CT_max_ was positively correlated with body mass in two species, *Dendropsophus minutus* (P = 0.014 and Adjusted R^2^ = 0.254) and *Noblella myrmecoides* (P = 0.012 and Adjusted R^2^ = 0.559); and CT_max_ was negatively correlated with body mass in one species, *Hamptophryne boliviana* (P = 0.027 and Adjusted R^2^ = 0.124). Additionally, at the intraspecific level, CT_min_ was not correlated with body size in three out of four species tested; CT_min_ was negatively correlated with body mass in one species, *Ctenophryne geayi* (P = 0.011 and Adjusted R^2^ = 0.635).

**Table A.** **Linear regression models between CT_max_ and body size estimated, and linear regression models between CT_min_ and body size.** Tests included only species represented by ≥ 8 measurements of CT_max_ or ≥ 5 measurements of CT_min_ in the dataset. The order of species listed follows the order provided in S1 Fig and S2 Fig. Bold font indicates significant values.

| **Model and Species tested** | **df** | **Intercept** | **F** | **_adj._ R^2^** | **P** |
| --- | --- | --- | --- | --- | --- |
| **CT_max_ ~ SVL** |  |  |  |  |  |
| *Rhinella margaritifera* | 7 | 33.52 | 1.236 | 0.029 | 0.303 |
| *Ameereha hahneli* | 11 | 42.44 | 0.955 | –0.004 | 0.350 |
| *Dendropsophus minutus* | 18 | 37.97 | 0.307 | –0.038 | 0.586 |
| *Scinax ictericus* | 10 | 40.43 | 0.068 | –0.093 | 0.800 |
| *Adenomera andreae* | 25 | 28.35 | 1.987 | 0.037 | 0.171 |
| *Edalorhina perezi* | 13 | 38.65 | 0.016 | –0.076 | 0.900 |
| *Chiasmocleis royi* | 21 | 38.27 | 0.090 | –0.043 | 0.768 |
| *Ctenophryne geayi* | 16 | 36.98 | 2.199 | 0.066 | 0.158 |
| *Hamptophryne boliviana* | 30 | 39.15 | 2.977 | 0.060 | 0.095 |
| *Noblella myrmecoides* | 7 | 25.05 | 6.019 | 0.386 | **0.044** |
| *Oreobates cruralis* | 6 | 29.01 | 1.795 | 0.102 | 0.229 |
| *Pristimantis carvalhoi* | 6 | 33.65 | 0.751 | –0.037 | 0.420 |
| *Pristimantis ockendeni* | 6 | 28.13 | 0.938 | –0.009 | 0.370 |
| *Pristimantis reichlei* | 35 | 30.31 | 1.674 | 0.018 | 0.204 |
| *Pristimantis toftae* | 15 | 33.19 | 0.321 | –0.044 | 0.579 |
| **CT_min_ ~ SVL** |  |  |  |  |  |
| *Ameereha hahneli* | 6 | 20.52 | 0.318 | –0.108 | 0.593 |
| *Edalorhina perezi* | 8 | 16.83 | 1.703 | 0.072 | 0.228 |
| *Ctenophryne geayi* | 6 | 13.14 | 3.436 | 0.258 | 0.113 |
| *Pristimantis reichlei* | 4 | 4.54 | 4.706 | 0.426 | 0.096 |
