# Supplementary material for: Thermal physiological traits in tropical lowland amphibians: Vulnerability to climate warming and cooling
Source: PLoS One. 2019 Aug 1;14(8):e0219759. doi: 10.1371/journal.pone.0219759 (PMC6675106; doi:10.1371/journal.pone.0219759)
Supplement: S2 File — (DOCX) [file pone.0219759.s002.docx]

**S2 File. Further methodological details on molecular phylogenetic analysis**. Our analysis included DNA sequences from species collected at our study site or from other areas in western Amazonia; most of these sequences were included in previous analyses [1–3], and we obtained new sequences from specimens collected at our main study site. We used sequences from three mitochondrial and two nuclear genes. The mitochondrial sequences included a section of the 16S rRNA gene, a section of the 12S rRNA gene, and the protein-coding gene cytochrome c oxidase subunit I (COI). The nuclear sequences included the protein-coding gene recombination-activating protein 1 (RAG1) and the nuclear protein-coding gene Tyrosinase precursor (Tyr). Extraction, amplification, and sequencing of DNA followed protocols described previously [4]. We deposited the newly obtained sequences in GenBank (S3 Supplementary Material). We used Geneious R6, version 6.1.8 [5] to align the sequences with the built-in multiple alignment program. We used a multispecies coalescent approach implemented in *BEAST 2 [6] to infer a Bayesian multilocus timetree of the focal taxa (please see additional details in the main body of the manuscript).

**References**

1. Pyron RA, Wiens JJ. A large-scale phylogeny of Amphibia including over 2800 species, and a revised classification of extant frogs, salamanders, and caecilians. Molecular Phylogenetics and Evolution. 2011; 61: 543–583.

2. Padial JM, Grant T, Frost DR. Molecular systematics of terraranas (Anura: Brachycephaloidea) with an assessment of the effects of alignment and optimality criteria. Zootaxa. 2014; 3825: 1–132.

3. Pyron RA. Biogeographic analysis reveals ancient continental vicariance and recent oceanic dispersal in amphibians. Systematic Biology. 2014; 63: 779–797.

4. von May R, Catenazzi A, Corl A, Santa-Cruz R, Carnaval AC, Moritz C. Divergence of thermal physiological traits in terrestrial breeding frogs along a tropical elevational gradient. Ecology and Evolution. 2017; 7: 3257–3267.

5. Biomatters. Geneious R6, version 6.1.5 (​http://www.geneious.com/), 2013.

6. Bouckaert R, Heled J, Kühnert D, Vaughan T, Wu C-H, Xie D, Suchard MA, Rambaut A, Drummond AJ. BEAST 2: A Software Platform for Bayesian Evolutionary Analysis. PLoS Computational Biology. 2014; 10: e1003537. doi:10.1371/journal.pcbi.1003537
