# Supplementary material for: Thermal physiological traits in tropical lowland amphibians: Vulnerability to climate warming and cooling
Source: PLoS One. 2019 Aug 1;14(8):e0219759. doi: 10.1371/journal.pone.0219759 (PMC6675106; doi:10.1371/journal.pone.0219759)
Supplement: S1 Table — Model fitting was done with the full dataset (50 species). Bold font indicates significant values. SVL = snout-vent length, BMI = body mass index, midpoint = elevational midpoint, Height = median height above the ground. (DOCX) [file pone.0219759.s003.docx]

**S1 Table.** **Results from phylogenetic generalized linear regression models to determine which factors best predict variation in CT_max_.** Model fitting was done with the full dataset (50 species). Bold font indicates significant values. SVL = snout-vent length, BMI = body mass index, midpoint = elevational midpoint, Height = median height above the ground.

| **Model** | **Estimate** | **λ** | **Coefficient** | **P-value** | **AIC** |
| --- | --- | --- | --- | --- | --- |
|  |  |  |  |  |  |
| CT_max_ ~ SVL | SVL | 0.82 | 0.155 | **<0.001** | 207.61 |
| CT_max_ ~ BMI | BMI | 0.89 | 1.471 | **<0.001** | 217.70 |
| CT_max_ ~ midpoint | Midpoint | 0.83 | <0.000 | 0.8559 | 234.70 |
| CT_max_ ~ height | height | 1.00 | 0.022 | **0.013** | 233.00 |
| CT_max_ ~ SVL + BMI |  | 0.83 |  |  | 209.43 |
|  | SVL |  | 0.141 | **0.002** |  |
|  | BMI |  | 0.200 | 0.682 |  |
| CT_max_ ~ SVL + midpoint |  | 0.83 |  |  | 209.60 |
|  | SVL |  | 0.155 | **<0.001** |  |
|  | midpoint |  | <0.000 | 0.942 |  |
| CT_max_ ~ SVL + height |  | 0.83 |  |  | 206.37 |
|  | SVL |  | 0.155 | **<0.001** |  |
|  | height |  | 0.016 | 0.082 |  |
| CT_max_ ~ BMI + midpoint |  | 0.90 |  |  | 219.60 |
|  | BMI |  | 1.486 | **<0.001** |  |
|  | midpoint |  | <0.000 | 0.752 |  |
| CT_max_ ~ BMI + height |  | 0.95 |  |  | 215.80 |
|  | BMI |  | 1.489 | **<0.001** |  |
|  | height |  | 0.018 | **0.043** |  |
| CT_max_ ~ midpoint + height |  | 1.00 |  |  | 235.00 |
|  | midpoint |  | <0.000 | 0.830 |  |
|  | height |  | 0.022 | 0.013 |  |
| CT_max_ ~ SVL + BMI + midpoint |  | 0.83 |  |  | 211.43 |
|  | SVL |  | 0.141 | **0.002** |  |
|  | BMI |  | 0.199 | 0.690 |  |
|  | midpoint |  | <0.000 | 0.993 |  |
| CT_max_ ~ SVL + BMI + height |  | 0.83 |  |  | 207.98 |
|  | SVL |  | 0.135 | **0.002** |  |
|  | BMI |  | 0.285 | 0.551 |  |
|  | height |  | 0.016 | 0.076 |  |
| CT_max_ ~ SVL + midpoint + height |  | 0.82 |  |  | 208.36 |
|  | SVL |  | 0.155 | **<0.001** |  |
|  | midpoint |  | <0.000 | 0.948 |  |
|  | height |  | 0.016 | 0.086 |  |
| CT_max_ ~ BMI + midpoint + height |  | 1.00 |  |  | 217.60 |
|  | BMI |  | 1.474 | **<0.001** |  |
|  | midpoint |  | –0.001 | 0.623 |  |
|  | height |  | 0.020 | **0.009** |  |
| CT_max_ ~ SVL+BMI+midpoint+height |  | 0.82 |  |  | 209.94 |
|  | SVL |  | 0.134 | **0.003** |  |
|  | BMI |  | 0.299 | 0.542 |  |
|  | midpoint |  | <0.000 | 0.864 |  |
|  | height |  | 0.106 | 0.078 |  |
