# Supplementary material for: Thermal physiological traits in tropical lowland amphibians: Vulnerability to climate warming and cooling
Source: PLoS One. 2019 Aug 1;14(8):e0219759. doi: 10.1371/journal.pone.0219759 (PMC6675106; doi:10.1371/journal.pone.0219759)
Supplement: S2 Table — Model fitting was done with the reduced dataset (37 species). Bold font indicates significant values. SVL = snout-vent length, BMI = body mass index, midpoint = elevational midpoint, Height = median height above the ground. (DOCX) [file pone.0219759.s004.docx]

**S2 Table.** **Results from phylogenetic generalized linear regression models to determine which factors best predict variation in CT_min_.** Model fitting was done with the reduced dataset (37 species). Bold font indicates significant values. SVL = snout-vent length, BMI = body mass index, midpoint = elevational midpoint, Height = median height above the ground.

| **Model** | **Estimate** | **λ** | **Coefficient** | **P-value** | **AIC** |
| --- | --- | --- | --- | --- | --- |
|  |  |  |  |  |  |
| CT_min_ ~ SVL |  | 0.77 | –0.129 | **<0.001** | 161.69 |
| CT_min_ ~ BMI |  | 0.82 | –0.231 | **<0.001** | 167.64 |
| CT_min_ ~ midpoint |  | 0.44 | –0.004 | 0.0806 | 170.95 |
| CT_min_ ~ height |  | 0.00 | –0.035 | **0.001** | 164.79 |
| CT_min_ ~ SVL + BMI |  | 0.61 |  |  | 163.42 |
|  | SVL |  | –0.154 | **0.013** |  |
|  | BMI |  | 0.436 | 0.514 |  |
| CT_min_ ~ SVL + midpoint |  | 0.69 |  |  | 161.00 |
|  | SVL |  | –0.118 | **<0.001** |  |
|  | midpoint |  | –0.003 | 0.116 |  |
| CT_min_ ~ SVL + height |  | 0.00 |  |  | 154.16 |
|  | SVL |  | –0.107 | **<0.001** |  |
|  | height |  | –0.033 | **<0.001** |  |
| CT_min_ ~ BMI + midpoint |  | 0.72 |  |  | 167.02 |
|  |  |  | –0.044 | **0.010** |  |
|  |  |  | –0.003 | 0.111 |  |
| CT_min_ ~ BMI + height |  | 0.00 |  |  | 153.98 |
|  | BMI |  | –1.251 | **<0.001** |  |
|  | height |  | –0.049 | **<0.001** |  |
| CT_min_ ~ midpoint + height |  | 0.24 |  |  | 165.00 |
|  | midpoint |  | –0.003 | 0.132 |  |
|  | height |  | –0.033 | **0.005** |  |
| CT_min_ ~ SVL + BMI + midpoint |  | 0.61 |  |  | 162.64 |
|  | SVL |  | –0.145 | **0.016** |  |
|  | BMI |  | 0.409 | 0.530 |  |
|  | midpoint |  | –0.003 | 0.118 |  |
| CT_min_ ~ SVL + BMI + height |  | 0.00 |  |  | 155.06 |
|  | SVL |  | –0.055 | 0.368 |  |
|  | BMI |  | –0.695 | 0.325 |  |
|  | height |  | –0.042 | **0.002** |  |
| CT_min_ ~ SVL + midpoint + height |  | 0.20 |  |  | 154.63 |
|  | SVL |  | –0.104 | **<0.001** |  |
|  | midpoint |  | –0.002 | 0.164 |  |
|  | height |  | –0.031 | **0.002** |  |
| CT_min_ ~ BMI + midpoint + height |  | 0.16 |  |  | 153.80 |
|  | BMI |  | –1.233 | **<0.001** |  |
|  | midpoint |  | –0.003 | 0.116 |  |
|  | height |  | –0.047 | **<0.001** |  |
| CT_min_ ~ SVL+BMI+midpoint+height |  | 0.16 |  |  | 155.20 |
|  | SVL |  | –0.044 | 0.473 |  |
|  | BMI |  | –0.788 | 0.266 |  |
|  | midpoint |  | –0.002 | 0.146 |  |
|  | height |  | 0.041 | **0.002** |  |
