# Supplementary figures and images for: Thermal physiological traits in tropical lowland amphibians: Vulnerability to climate warming and cooling
Source: PLoS One. 2019 Aug 1;14(8):e0219759. doi: 10.1371/journal.pone.0219759 (PMC6675106; doi:10.1371/journal.pone.0219759)

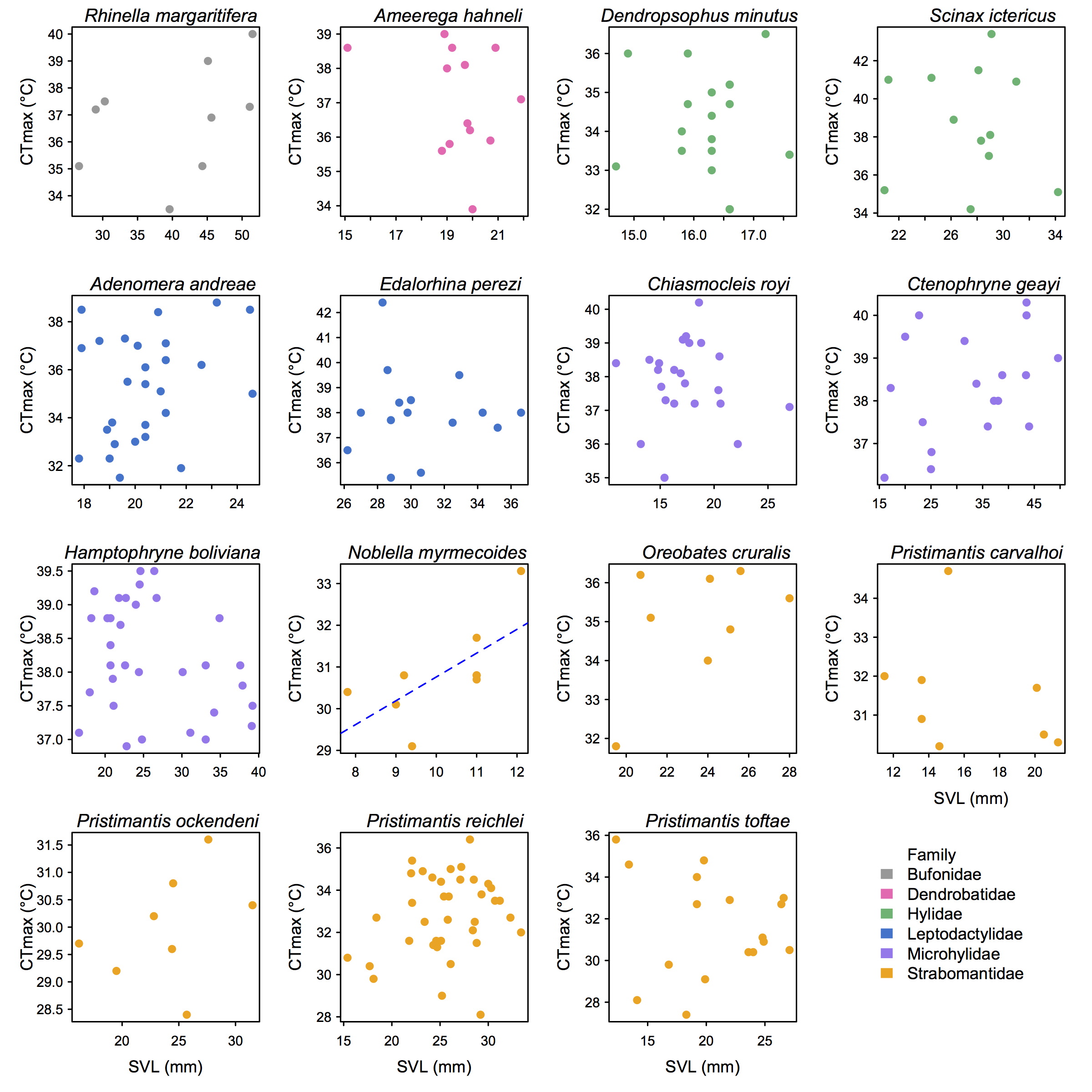

Supplement: S1 Fig — At the intraspecific level, CTmax was not correlated with body size in all species tested except Noblella myrmecoides. (TIFF) [file pone.0219759.s005.tiff]

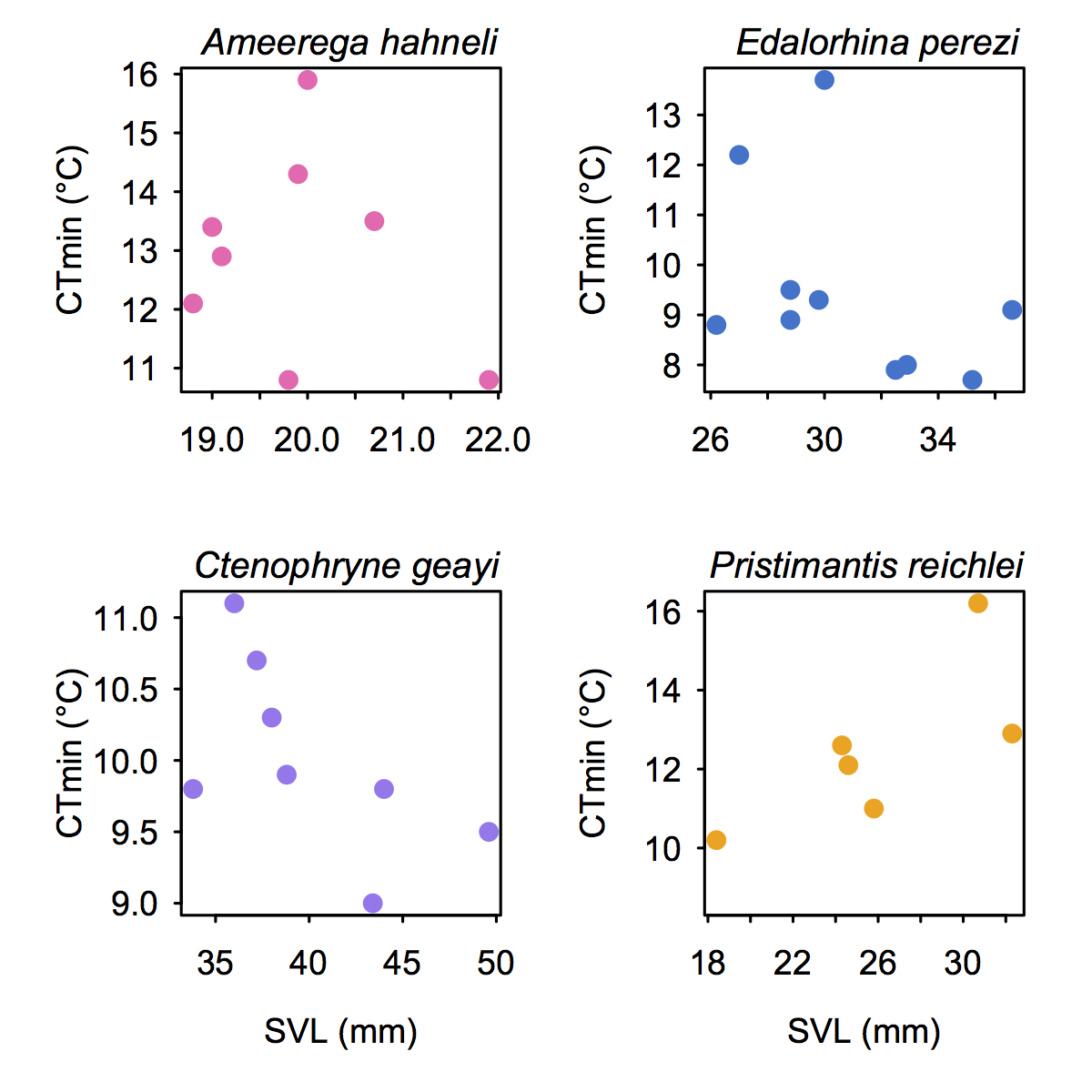

Supplement: S2 Fig — At the intraspecific level, CTmin was not correlated with body size. (TIFF) [file pone.0219759.s006.tiff]

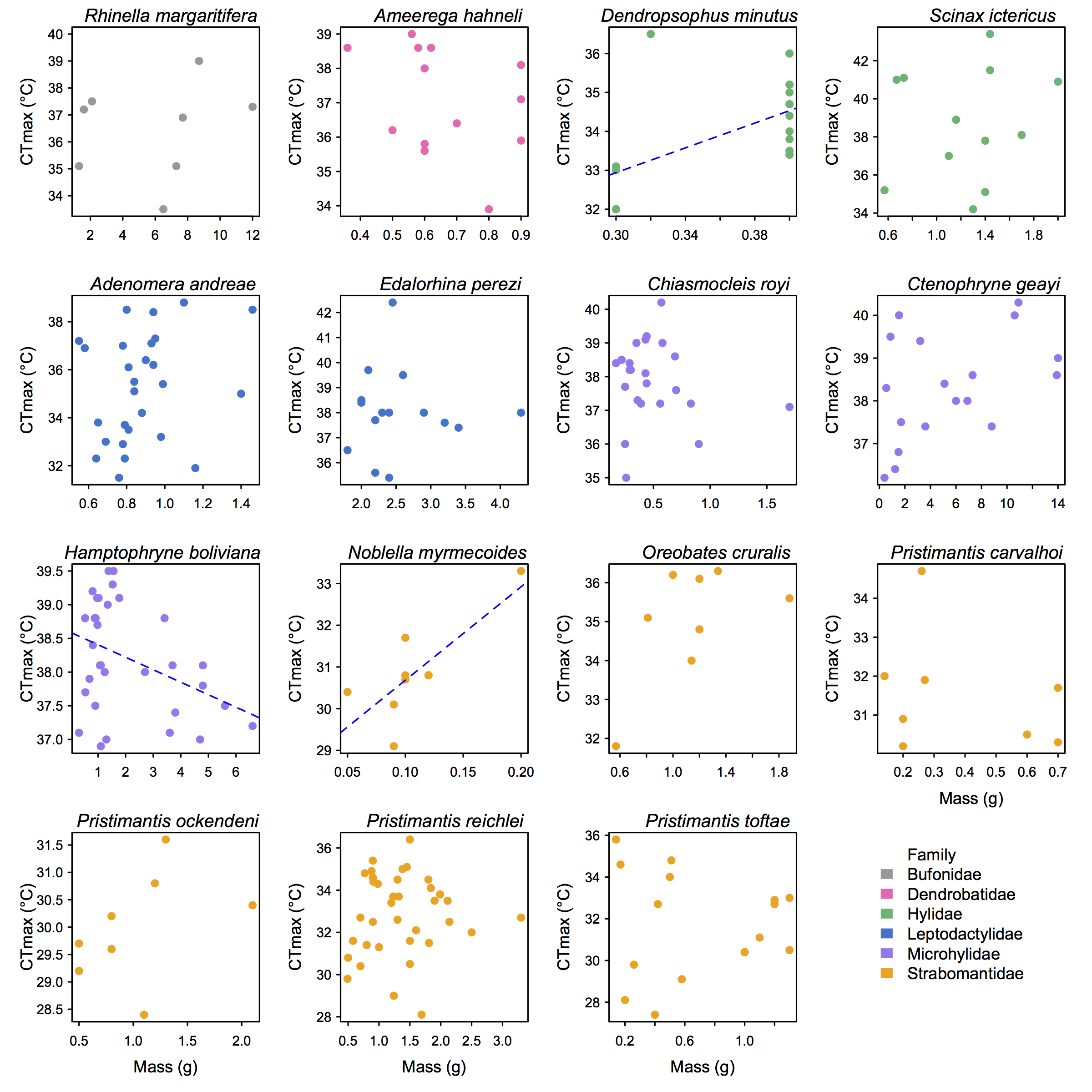

Supplement: S3 Fig — At the intraspecific level, CTmax was not correlated with body mass in most species tested. (TIFF) [file pone.0219759.s007.tiff]

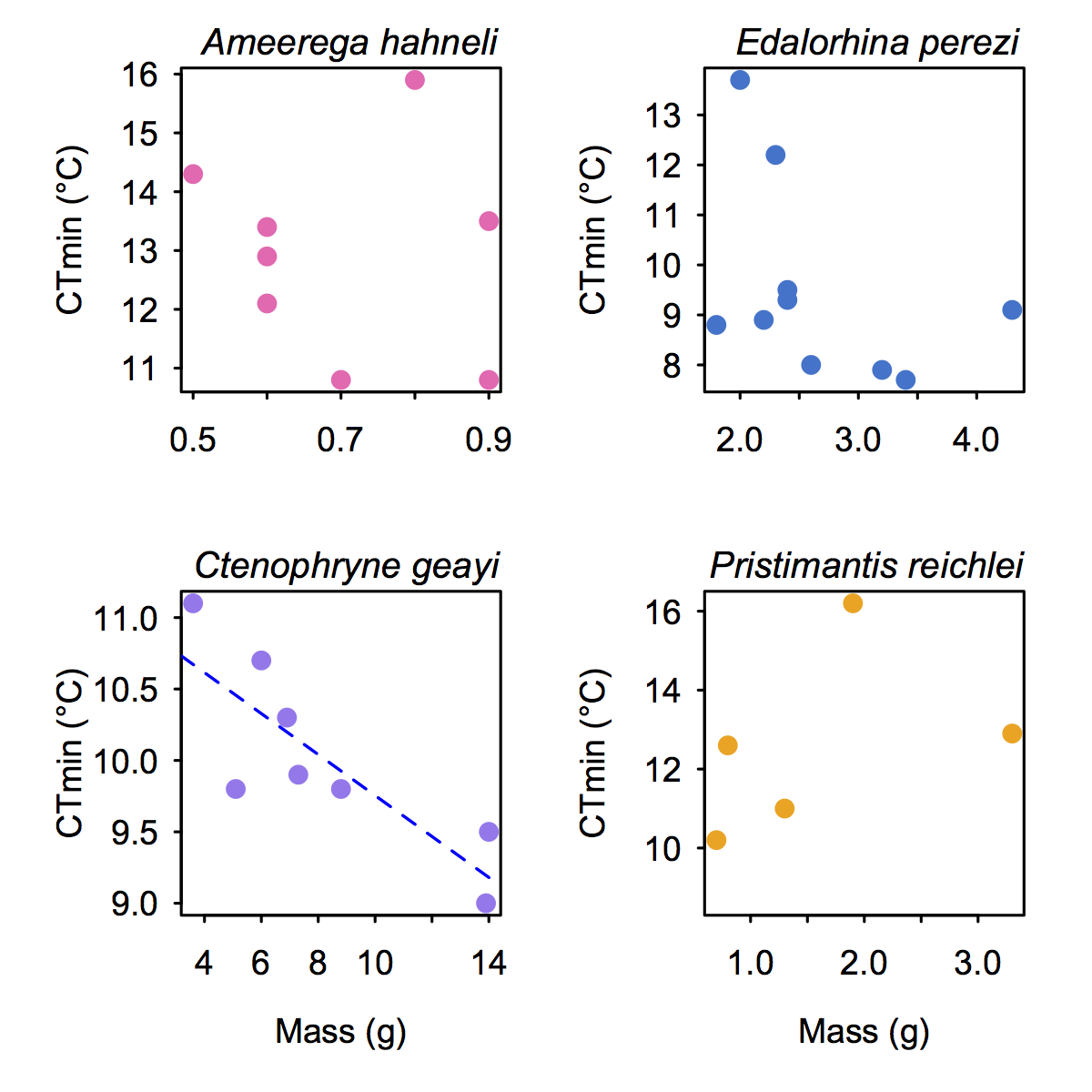

Supplement: S4 Fig — At the intraspecific level, CTmin was not correlated with body mass in most species tested. (TIFF) [file pone.0219759.s008.tiff]

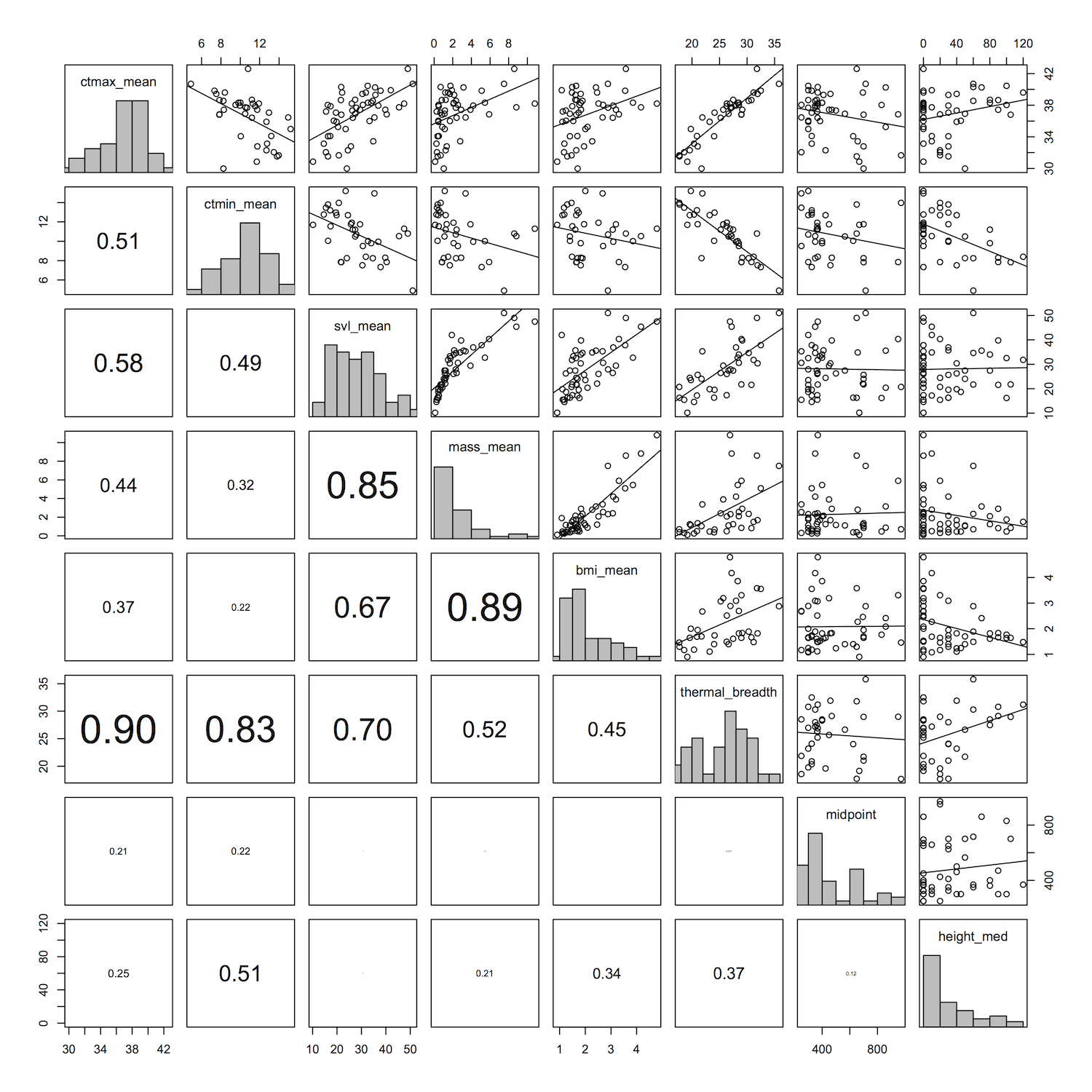

Supplement: S5 Fig — (TIFF) [file pone.0219759.s009.tiff]

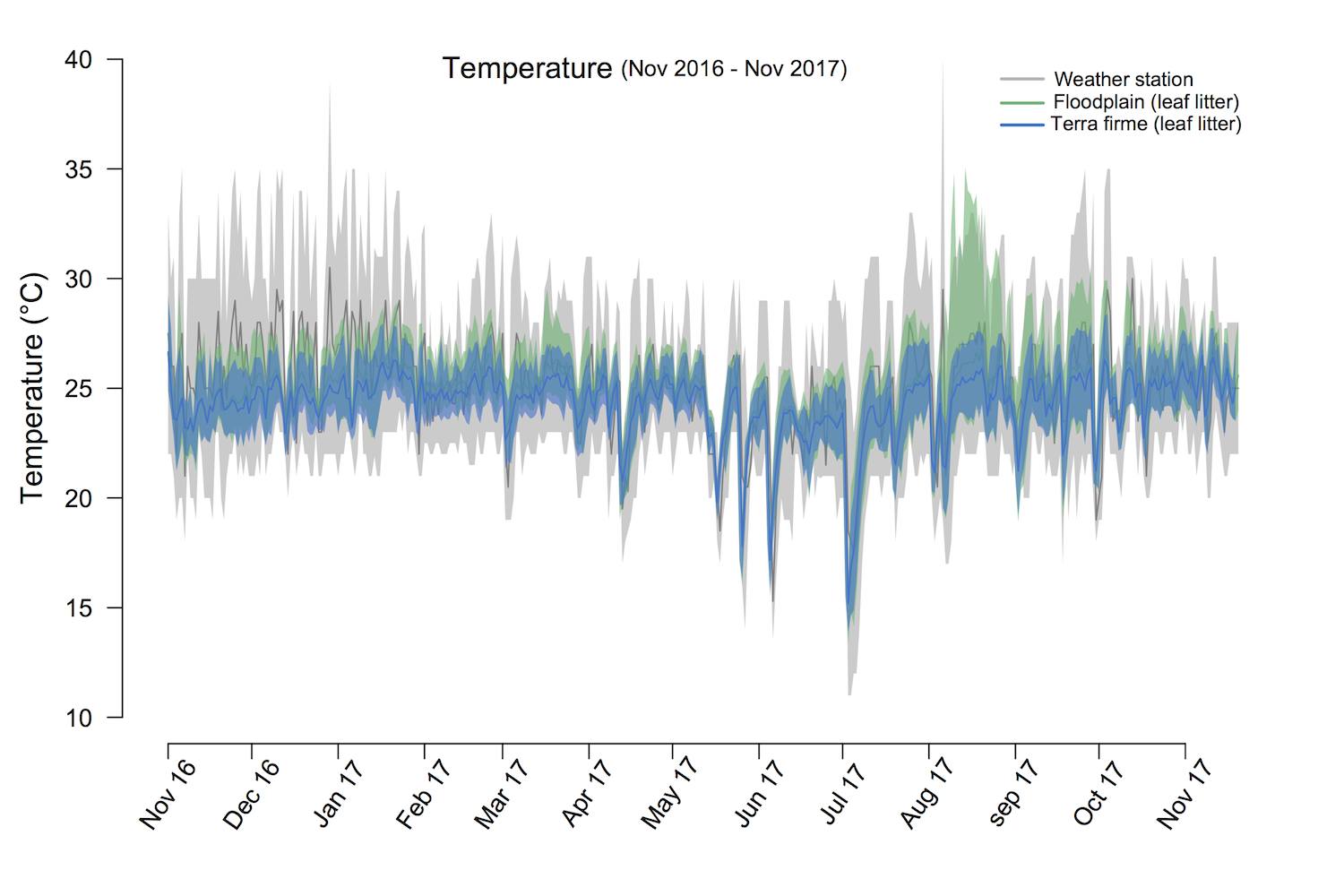

Supplement: S6 Fig — Daily temperatures recorded from 16 November 2016 to 5 December 2017 (385 days) in two forest types at Los Amigos Biological Station, Peru. Weather station data are shown in gray; temperature data collected in the leaf litter are shown in green (floodplain forest) and blue (terra firme forest). In each case, the maximum and minimum temperatures delimited the polygons and the line in the middle represents the mean temperature. (TIFF) [file pone.0219759.s010.tiff]
