# Supplementary material for: Thermal physiological traits in tropical lowland amphibians: Vulnerability to climate warming and cooling
Source: PLoS One. 2019 Aug 1;14(8):e0219759. doi: 10.1371/journal.pone.0219759 (PMC6675106; doi:10.1371/journal.pone.0219759)
Supplement: S2 Appendix — (DOCX) [file pone.0219759.s012.docx]

**S2 Appendix.** **Voucher numbers and GenBank accession numbers for the taxa and genes sampled in this study.** Numbers in bold are new sequences generated in this study.

| **Taxon** | **Voucher_Nbr** | **16S** | **12S** | **RAG1** | **Tyr** |
| --- | --- | --- | --- | --- | --- |
| *Adenomera andreae* | MUSA 7017 | **MN172508** | NA | NA | NA |
| *Allobates conspicuus* | OMNH 35997 | DQ502135 | DQ502135 | DQ503348 | NA |
| *Allobates femoralis* | MJH 7354 | DQ502117 | EU342537 | DQ503327 | DQ503156 |
| *Allobates trilineatus* | MJH 7477 | DQ502118 | DQ501998 | DQ503290 | NA |
| *Allobates trilineatus* | MUSA 6304 | **MN172509** | NA | NA | NA |
| *Amazophrynella javierbustamantei* | MUSA 6994 | **MN172510** | NA | NA | NA |
| *Ameerega hahneli* | QCAZ 13325 | AY364573 | EU342622 | NA | DQ503174 |
| *Ameerega trivittata* | LSUMZ 13693 | EU342633 | EU342634 | DQ503302 | DQ503146 |
| *Boana cinerascens* | AMNH-A 164105 | AY549336 | NA | NA | NA |
| *Boana geographica* | MHNG 2607.01 | **MN172520** | NA | NA | NA |
| *Boana lanciformis* | KU 202724 | AY326054 | AY326054 | NA | AY844081 |
| *Boana punctata* | CORBIDI 11846 | **MN172521** | NA | NA | NA |
| *Boana* sp. G | CORBIDI 11852 | **MN172519** | NA | NA | NA |
| *Chiasmocleis bassleri* | KU 222104 | JF836990 | FJ752367 | NA | NA |
| *Chiasmocleis royi* | KU 215540 | KC180059 | JF836938 | NA | NA |
| *Ctenophryne geayi* | MUSA 6324 | **MN172511** | NA | NA | NA |
| *Ctenophryne geayi* | AMNH A166444 | DQ283383 | DQ283383 | NA | NA |
| *Dendropsophus leucophyllatus* | R.Bran 95253 | AF308097 | DQ380360 | NA | NA |
| *Dendropsophus minutus* | CORBIDI 11850 | **MN172513** | NA | NA | NA |
| *Dendropsophus koechlini* | MUSA 6321 | **MN172512** | NA | NA | NA |
| *Dendropsophus kamagarini* | CORBIDI 17477 | **MN172514** | NA | NA | NA |
| *Dendropsophus kamagarini* | AMNH-A 139315 | AY843652 | AY843652 | AY844440 | AY844097 |
| *Dendropsophus schubarti* | MUSA 6310 | **MN172515** | NA | NA | NA |
| *Dendropsophus triangulum* | WED 54094 | AY326053 | AY326053 | AY844464 | AY844122 |
| *Dendropsophus triangulum* | MUSA 6311 | **MN172516** | NA | NA | NA |
| *Edalorhina perezi* | MJH 7082 | AY843585 | AY843585 | NA | NA |
| *Elachistocleis muiraquitan* | MUSA 7018 | **MN172517** | NA | NA | NA |
| *Engystomops freibergi* | FG SCM299 | JN970383 | AF058962 | NA | NA |
| *Hamptophryne boliviana* | MUSA 6309 | **MN172518** | NA | NA | NA |
| *Leptodactylus bolivianus* | JDL 26591 | HQ232846 | NA | NA | NA |
| *Leptodactylus didymus* | USNM 268970 | AY948957 | AY819346 | NA | NA |
| *Leptodactylus leptodactyloides* | MZUSP 70969 | AY943236 | AY943223 | NA | NA |
| *Leptodactylus petersii* | KU 218194 | KM091608 | NA | NA | NA |
| *Lithodytes lineatus* | USP 968438 | AY326012 | AY326012 | NA | AY844129 |
| *Noblella myrmecoides* | RvM 3.12 | KY652644 | NA | KY672962 | KY681065 |
| *Oreobates cruralis* | MUSM 33248 | KY652647 | NA | KY672963 | KY681068 |
| *Oreobates quixensis* | KU 218150 | DQ679380 | AY819344 |  |  |
| *Pristimantis buccinator* | MUSM 33269 | KY652650 | NA | KY672966 | KY681071 |
| *Pristimantis carvalhoi* | CORBIDI 16294 | KY652651 | NA | KY672967 | KY681072 |
| *Pristimantis ockendeni* | RvM 5.12 | KY652654 | NA | KY672970 | KY681075 |
| *Pristimantis reichlei* | CORBIDI 16219 | KY652657 | NA | KY672972 | KY681078 |
| *Pristimantis toftae* | AC 107.07 | KY652659 | NA | KY672974 | KY681080 |
| *Osteocephalus buckleyi* | MUSA 6977 | **MN172522** | NA | NA | NA |
| *Osteocephalus buckleyi* | MUSA 6995 | **MN172523** | NA | NA | NA |
| *Phyllomedusa camba* | MUSA 7001 | **MN172524** | NA | NA | NA |
| *Phyllomedusa camba* | MUSA 7013 | **MN172525** | NA | NA | NA |
| *Phyllomedusa vaillantii* | AMNHA 1662888 | AY549363 | AY549363 | AY844498 | AY844158 |
| *Pristimantis fenestratus* | MHNSM 9298 | EF493703 | FJ438809 | NA | NA |
| *Ranitomeya sirensis* | NA | AF482794 | AF482779 | EU325913 | NA |
| *Rhinella margaritifera* | AGG 172 | AF375514 | AY819331 | NA | EF364358 |
| *Rhinella marina* | KU 205236 | AY325994 | AY325994 | DQ158393 | NA |
| *Scarthyla goinorum* | QULC 2340 | AY843752 | AY843752 | AY844514 | NA |
| *Scarthyla goinorum* | MUSA 6319 | **MN172526** | NA | NA | NA |
| *Scinax ictericus* | MUSA 6322 | **MN172527** | NA | NA | NA |
| *Scinax ruber* | KU 207622 | AY326034 | AY326034 | AY844521 | AY844181 |
| *Sphaenorhynchus lacteus* | USNM 268930 | AY549367 | AY819394 | AY844527 | AY844188 |
| *Sphaenorhynchus lacteus* | MUSA 6330 | **MN172528** | NA | NA | NA |
| *Trachycephalus typhonius* | VUB 987 | FJ882779 | DQ347027 | EU034147 | DQ347161 |
